# A Semi-Automatic Method on a Small Italian Sample for Estimating Sex Based on the Shape of the Crown of the Maxillary Posterior Teeth

**DOI:** 10.3390/healthcare11060845

**Published:** 2023-03-13

**Authors:** Ilenia Bianchi, Giorgio Oliva, Giulia Vitale, Beatrice Bellugi, Giorgio Bertana, Martina Focardi, Simone Grassi, Domenico Dalessandri, Vilma Pinchi

**Affiliations:** 1 Laboratory of Personal Identification and Forensic Morphology, Department of Health Sciences, University of Florence, Largo Brambilla 3, 50134 Florence, Italy; 2 Department of Law, University of Macerata, 62100 Macerata, Italy; 3 Department of Medical and Surgical Specialties, Radiological Sciences and Public Health, School of Dentistry, University of Brescia, Piazzale Spedali Civili 1, 25123 Brescia, Italy

**Keywords:** geometric morphometric analysis, artificial neural network, sex estimation, forensic odontology, dental sexual dimorphism, human identification

## Abstract

Teeth are known to be reliable substrates for human identification and are endowed with significant sexual dimorphism not only in the size but also in the shape of the crowns. In the preliminary phase of our study (already published in 2021), a novel sex estimation method based on dental morphometric geometric (GMA) analysis combined with the artificial neural network (ANN) was developed and validated on a single dental element (first upper premolar) with an accuracy rate of 80%. This study aims to experiment and validate the combination of GMA–ANN on the upper first and second left premolars and the upper left first molar to obtain a reliable classification model based on the sexual dimorphic traits of multiple maxillary teeth of Caucasian Italian adults (115 males and 115 females). A general procrustes superimposition (GPS) and principal component analysis (PCA) were performed to study the shape variance between the sexes and to reduce the data variations. The “set-aside” approach was used to validate the accuracy of the proposed ANN. As the main findings, the proposed method correctly classified 94% of females and 68% of males from the test sample and the overall accuracy gained was 82%, higher than the odontometric methods that similarly consider multiple teeth. The shape variation between male and female premolars represents the best dimorphic feature compared with the first upper molar. Future research could overcome some limitations by considering a larger sample of subjects and experimenting with the use of computer vision for automatic landmark positioning and should verify the present evidence in samples with different ancestry.

## 1. Introduction

Sex estimation is a pivotal step in any identification process of unidentified or unrecognizable corpses, but as happens in some forensic cases, the recovered human remains often offer insufficient evidence for a conclusive sex identification [1,2]. Several techniques and substrates with different degrees of accuracy can be applied in this forensic field, since the reliability of the different methods largely depends on the available elements useful for the analysis and their integrity over time [2]. The main approaches for the sex estimation of human remains are based on metric, non-metric (or morphological), and molecular methods [2]. Despite the strong reliance of the forensic community on molecular methods, which in many cases allow the lowest rate of errors for a conclusive and reliable result [3], a recent review published by Interpol revealed that the application of DNA in criminal investigations is limited in practice to only a few countries [4], whilst metric and morphological methods are still the most used techniques [2]. On one hand, DNA analysis is often expensive in terms of costs and tissue loss. On the other hand, in a forensic context which considers skeletal remains, the reliability of the genetic results could be compromised by the degradation rate of the nucleic acids, the very small amount of available nuclear DNA, the presence of enzyme inhibitors in the DNA extracts, the possible weak amplification of the Y band, and the risk of contamination during sample collection and/or handling [5]. Nonetheless, the metric and morphological methods normally used on skeletal remains offer limited applicability in cases of mass disasters where corpses could be dismembered and mutilated, in adolescent subjects before the achievement of sexual development, in burnt bodies because of the shrinkage of bones, and in isolated skulls, jaws, or recovered teeth [2].

Within this forensic framework, teeth can be of pivotal value for sex identification because of their characteristics of high resistance to environmental factors due to the hardness of tissues, and the significative sexual dimorphism both in size and shape that can be applied in the most critical cases instead of the classic skeletal or genetic methods, especially in cases of mass disaster, isolated skull or jaw recovery, charred bodies, archaeological findings, and young pre-puberal skeletons [1,6,7,8]. Moreover, dental pulp seems to be an excellent tool for DNA extraction for forensic purposes since it is stable for longer and less contaminated than other sources of blood due to the hard tissue protection and the localization inside the oral cavity, which is richly hydrated and very resistant even to high temperatures [9].

The main odontological sex estimation approaches include non-invasive techniques, which are largely indicated especially in cases of human remains, skeletal fragments, and isolated skulls or jaws, where the tissue damage represents a major risk of loss of information for identification purpose. In particular, they can be simplified in metric methods, which quantify the difference in dental size, and non-metric or morphological methods, which analyse the anatomical variations of the dental shape between males and females [1,10,11].

There is shared agreement in the previous scientific evidence that the two-dimensional measurements of metric methods have some major limitations in terms of their forensic applications since the accuracy of these methods considerably decreases when incomplete dentition is analysed and the obtained results are not satisfactorily consistent to suggest the use of odontometrics as unique tool for sex estimation, whilst morphological methods can offer only a qualitative evaluation largely affected by the operator’s subjectivity and the variability of dental anomalies [1,10,11,12,13,14,15,16,17,18,19,20,21,22,23,24,25,26,27,28,29,30]. 

Therefore, our research aims to test techniques which are able to quantify the three-dimensional morphological characteristics of teeth. Some novel studies have considered the geometric morphometric analysis (GMA) [19,25,30,31,32], which allows the analytical study of the three-dimensional dental crown surfaces based on fixed landmarks and sliding semi-landmarks, quantifying the morphological characteristics and separately measuring both the shape and size differences between the sexes. Especially in archaeological fields, dental evidence is easily available even in the presence of largely compromised skeletal remains, and greater attention has been paid to identification methods based on geometric morphometric analysis [6]. However, GMA is a complex measurement approach that produces vectors that are not easy to interpret as standard linear measurements and not useful for a reliable sex classification.

To cope with these relevant limitations of the applicability of GMA, Oliva et al., 2021 [30] developed and validated a promising sex classification method based on the combination of GMA with artificial intelligence systems (artificial neural networks—ANNs). The pilot method [30] allowed a correct classification of 80% of the test sample analysing the crown morphology exclusively of the first upper left first premolar. These results were more reliable than those of Yong’s study, which applied GMA not combined with ANN with an accuracy mainly below 70% and concluded that the occlusal shapes of male and female premolars are not endowed with significant sexual dimorphism [25].

This study aims to validate on a larger scale and on several teeth the results gained by the pilot research of Oliva et al. since no similar studies are available in the literature. Hence, the combination of the GMA–ANN technique has been experimented with and validated on upper left first and second premolars and upper left first molars. The endpoint is to obtain a reliable and accurate classification model based on the sexual dimorphic traits of maxillary premolars and first molars, since the previous literature [13,15,16,17,18,20] has demonstrated that the combination of more teeth improves the accuracy of sex estimation.

## 2. Materials and Methods

A total of 230 dental scans from Caucasian Italian adults (115 males and 115 females, mean age: 35 ± 8 years) who underwent dental care for orthodontic clinical reasons were analysed.

All patients gave their informed consent for the anonymous use of their intraoral scans for the study, and only sex and age data were registered in accordance with ethics principles.

The inclusion criteria were patients without missing teeth, dental decay, pathologic anomalies of enamel and enamel/dentin, significant wear, or a remarkable medical history.

The exclusion criteria were patients who did not meet one or more of the inclusion criteria and gross irregularity of the 3D scans.

Only the upper teeth of the left side were analysed in this study: the maxillary first and second premolars and maxillary first molar. These teeth were selected on the basis of previous literature, which demonstrated that the teeth of the upper arch have greater sexual dimorphism [15,18] and, in particular, the maxillary premolars were shown to have the highest sexual dimorphism [28]. This feature tends to be preserved during the lifetime since the first premolar is the tooth least affected by physiological wear [33].

The first molar was added in this study since it was shown to be the most useful tooth, together with the canine, in odontometric methods for sex estimation [10,34]. Moreover, first molars are available in pre-adolescent children, whose skeletons or remains are hard to classify by sex [20,35].

Additionally, only one side was analysed considering the symmetry of dental traits between right- and left-side measurements as demonstrated by previous research [23,36].

The landmark digitations of the three different teeth surfaces were performed by the same operator experienced in this kind of analysis.

The occlusal morphology of the teeth was studied by means of 3D geometric morphometrics. Landmark digitization was performed using Viewbox 4.0 software (dHAL software; Kifissia, Greece).

**Figure 1:**
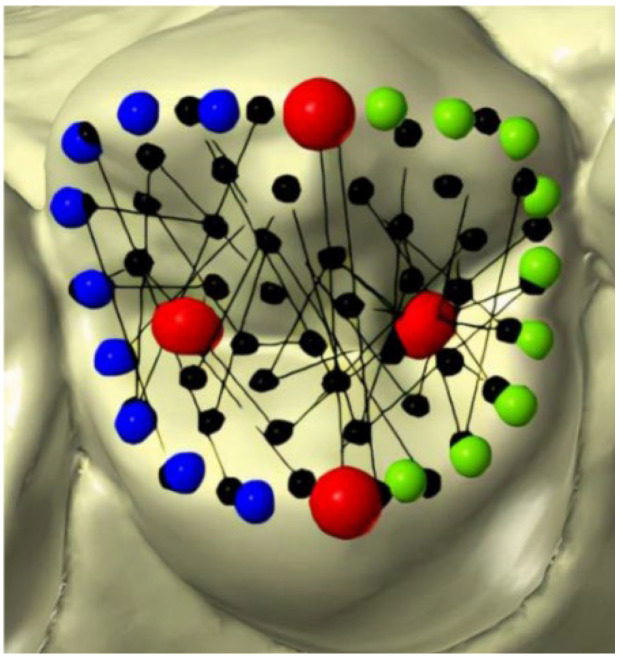
Distribution of landmarks (red points) and semilandmarks (blue, green, and black points) on the premolar occlusal surface.

**Figure 2:**
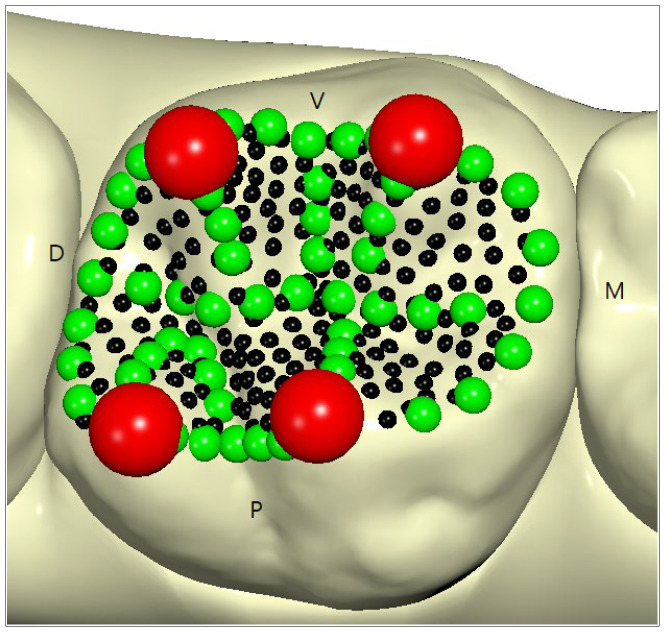
Distribution of landmarks (red points) and semilandmarks (green and black points) on the molar occlusal surface.

The protocol developed by Oliva et al. [30] was used for landmark placement in the premolars (Figure 1), whilst for the molars we followed the protocol of Polychronis et al. [19] (Figure 2).

Some steps of the mapping were operator-dependent, while others were developed in an automated way by the software (Viewbox 4.0, dHAL software; Kifissia, Greece). The operator proceeded with the placement of some reference points (cusp landmarks and curve semilandmarks); the remaining surface semilandmarks were placed automatically by the software using thin plate models previously developed by an orthodontic professional experienced in this skill (Figure 1 and Figure 2). In particular, the operator placed the fixed landmarks on the principal cusps and fossae of both the premolars and molars, and placed the curve semilandmarks on the principal ridges. Then, the software automatically placed equally spaced semi-landmarks along each curve and a *thin plate spline* transformation was used to automatically transpose the surface semilandmarks to map the entire occlusal crown morphology.

To avoid any possible variability of the manual placement, the model allows for the orientation of the 3D image in order to perform a double check of the positions of reference points during digitation: a first positioning according to the side view and then an adjustment in the occlusal view.

The semi-automated steps for the premolar mapping followed the scheme proposed in the study of Oliva et al. [30].

1. Four fixed landmarks were manually placed on each tooth: the buccal and lingual cusp tips and the mesial and distal fossae. All the landmarks were initially projected from the occlusal view and double checked by rotating the models (red points).
2. Nine semi-landmarks were placed manually to identify major ridges and to delimitate the occlusal circumference. To accomplish this, two curves were drawn over the mesial and distal ridges, respectively, connecting the buccal and lingual cusp tips. The software automatically placed equally spaced semi-landmarks along each curve (blue and green points).
3. Fifty surface semi-landmarks were automatically transposed to all the specimens using *thin plate spline* transformation (black points).
4. The curve and surface semi-landmarks were slid to minimize the bending energy between each premolar configuration and the reference specimen. Then, the semilandmarks were automatically re-projected six times on their curves or surfaces.

For the upper first molars, the positioning of landmarks and semilandmarks was developed ex novo according to the protocol of Polychronis et al. [19]:

1. Four fixed landmarks were manually placed in correspondence with the mesio-palatine, disto-palatine, disto-buccal, and mesio-buccal cusps. All the landmarks were initially projected from an occlusal view and double-checked by rotating the models (four red dots).
2. Fifty-one semilandmarks were placed manually to identify the mesial and distal marginal ridges, the palatal ridge, and the buccal ridge. Then, in sequence, the central sulcus, buccal sulcus, and palatal sulcus. Finally, all the cusp ridges were analysed—the mesio-palatal, mesio-buccal, disto-palatal, and disto-buccal—reaching a total of 51 semi-landmarks on the curves (green dots).
3. A total of 210 surface semi-landmarks were then automatically added using the configuration adopted with the *thin plate spline* transformation (black dots).

To study the shape of the teeth, a general procrustes superimposition (GPS) was then performed according to the method applied by Oliva et al. [30]. Size differences were defined by centroid size; the distance between landmark configurations in the shape space was used to measure the shape variance in the whole sample.

To assess the repeatability of the morphometric method and the operator variability in positioning the reference points, the intra- and interrater agreements were calculated from the GPS results based on the second round of measurements carried out after 30 days on 30 randomly selected scans and provided, respectively, by the same operator and a second orthodontist.

After the creation of a covariance matrix to represent the variation of each variable with respect to the others, a principal component analysis (PCA) was performed, and the first 30 components were considered for each tooth. Together with these components, the natural logarithm of the centroid size was also considered as a predictor. A total of 93 predictors were used to create an artificial neural network (ANN) with the aim of classifying subjects by gender.

The “set-aside” approach was used to assess the accuracy of the proposed ANN [25,37] by diving the sample into two subgroups: the training sample (174 scans, 84 F and 90 M) used to build the ANN and the test sample (56 scans, 31 F e 25 M), on which the obtained ANN was applied in order to measure its performance in predicting sex.

A dedicated ANN was applied in which all the neurons had sigmoidal activation and the optimization was performed using stochastic gradient descent. The appropriate number of hidden neurons was determined using the 10-K cross-validation method to maximize the ANN’s accuracy.

The performance of the obtained ANN in predicting sex was measured in the test sample by using a confounding matrix with “female” as the positive value.

All the analyses were carried out using the R package caret. In particular, this package uses the neural net package protocols for artificial neural network training and optimization.

**Figure 3:**
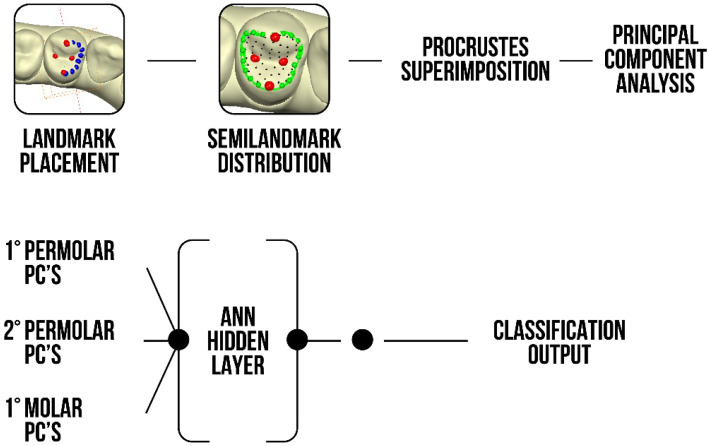
Outline of the study starting from the positioning of the landmarks and semi-landmarks up to the ANN output. PC: principal components.

The entire outline of the study is summarized in Figure 3.

## 3. Results

The intra- and interrater agreements were evaluated based on the procrustes distance obtained from the first and the second round of digitations carried out on 30 randomly selected scans, and the respective results were 0.95% and 0.98%. Only 1% of the total shape variance was found to be due to measurement errors.

The difference in centroid size according to sex was statistically significant, with a *p* value less than 0.05, for all the teeth.

**Figure 4:**
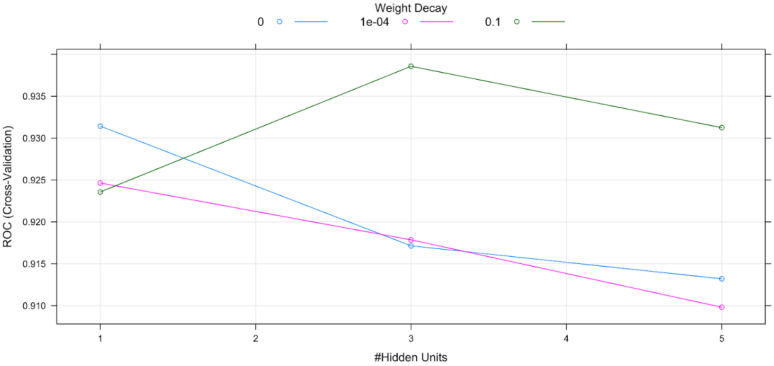
10-k cross-validation method used to optimize the ANN parameters.

For the realization of the ANN, the number of hidden layers was three and the decay rate was 0.1. Figure 4 shows the 10-k cross-validation method used to optimize the ANN parameters.

**Figure 5:**
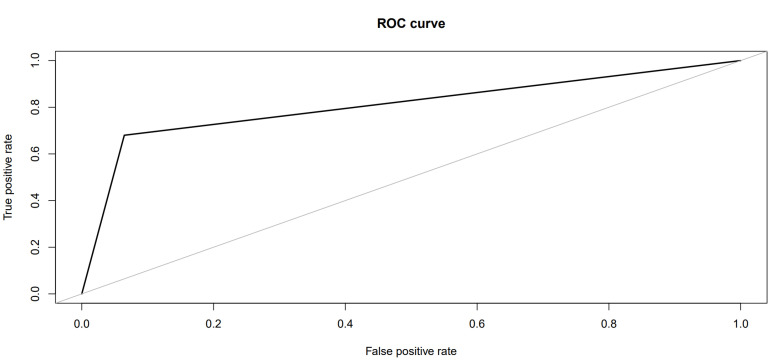
The ROC curve shows the performance of the prediction model on the control group.

A ROC curve (Figure 5) was performed on the results of the test sample. The curve showed the performance of the described algorithm in contrast to a random allocation.

The confusion matrix was performed and reported in Table 1.

The overall accuracy in classifying subjects by sex was found to be 94% for the training sample and 82% for the test sample.

“Female” was randomly selected as the “positive” value. The post-test probabilities (Se, Sp, PPV, and NPV) revealed that females showed a higher probability of being correctly positively classified as females (Se = 94%), whereas males showed a higher probability of not being misclassified as females (NPV = 89%).

From the PCA analysis, it was observed that the first 50 components (significant predictors) represent 94.4% of the information present overall in the sample, sufficient to avoid the hyper-dimensionality of the data (Table 2).

The results in Table 2 show that the highest number of significant predictors for sex classification belongs to the premolars in comparison to the first molar and that the weightiest predictor in terms of shape variation between males and females is the principal component number 4 belonging to the upper second premolar (SP004).

## 4. Discussion

Teeth are excellent substrates for research in many forensic fields since they are mineralized tissues characterized by a high resistance to environmental factors [38]. They are well-known as valuable sources of evidence in the identification process, both as primary identifiers for unknown dead bodies [39] and contributing to the estimation of the biological profile of human remains [40]. As regards the latter point, sex estimation is one of the pillar diagnoses that should be achieved in a forensic context. As can happen in mass disasters or in some forensic or archaeological cases, e.g., the recovery of isolated skulls or jaws, charred corpses, and young pre-puberal skeletons, the recovered human remains can offer insufficient evidence for a conclusive sex identification based on common skeletal and genetic analysis. Dental size and traits have been shown to have significative variations between male and female subjects [6,7,8,12,13,14,15,16,17,18,19,20,21,22,23,24,25,26,27,28,29,30] and odontometric methods in particular have been experimented with in different populations for sex diagnosis. However, odontometrics requires the analysis of numerous dental elements to obtain high levels of reliability (Table 3), thereby the method applicability depends on the kind and the number of retrieved teeth from the human remains. Despite several studies which have helped to refine the use of traditional morphometric methods on various skeleton sections to validate sexual dimorphism by shape variation between males and females [10], very few authors have considered applying GMA to the study of dental sexual dimorphism. Some authors concluded that GMA was a complex technique that only gained somewhat irrelevant improvements in accuracy compared to easier odontometric techniques for sex diagnosis [19,25]. Oliva et al. [20] overcame the computational complexity by combining GMA with an ANN for the study of only the first left premolar, achieving an high accuracy in classifying subjects by sex.

So, the aim of the study was to assess the use of the GMA–ANN technique for sex estimation on a predictive model based on several teeth, since no similar studies are available in the literature.

As a first result, GMA performed with the use of ANN on dental scans was confirmed to be a highly reproducible method, since the intra- and interrater agreement was 0.95% and 0.98%, respectively. This percentage is the same as that found by Oliva et al. [30] and it is within the acceptable limits.

Based on the number of the analysed sample, a principal component analysis (PCA) was used to obtain a reduction of the data and to study the variance of the procrustes superimposition (Figure 3). This choice of method, in comparison with the use of PLS (partial least square) analysis [30], enabled us to consider 30 components of each tooth together with the natural logarithm of the centroid size for a total of 93 predictors which were used to create the new ANN with the aim of classifying subjects by sex. PCA statistics should be always applied for a large sample, due to its better performance in complex factor analysis in terms of limiting the loss of information, as the GMA implies.

Using PCA predictors, the ANN was developed (the number of hidden layers was three with decay rate of 0.1) and applied to the test sample to cope with the required computations and analysis. Comparing the confusion matrix results of the ANN (Table 1) with the results gained from our previous research [30] (Table 3, n. 10), we found a small improvement in the sex classification accuracy (82% in test sample) using three different occlusal surfaces (first and second upper left premolars and first upper left molar) compared to Oliva’s technique based only on the first upper left premolar (accuracy 80%). This finding could be explained by the low sexual dimorphism of the first upper molar demonstrated by the PCA results which revealed that most significant predictors for sex classification belong to the premolars (Table 2) in line with the previous research of Oliva et al. [30]. So, premolars were shown to have a high degree of sexual dimorphism in terms of the shape variation between males and females compared to the first molar in the studied Italian sample (Table 1). However, this result was unexpected given the evidence gained from previous literature based on classic metric methods, which reported that the larger the number of the enrolled teeth, the higher the accuracy of sex estimation was (Table 3, n. 1–8).

Nevertheless, the method can be considered reliable as it gained high values of sensitivity (94%) and post-test probability (NPV 89%) in the test sample (Table 1). Taking into account that “female” was randomly selected as the “positive” value, the discriminating capabilities of the studied ANN allowed a very high probability of correctly positively classifying females (Se = real positives), whereas males showed a lower probability of being correctly negatively classified with a specificity of 68% (Sp = real negatives). The post-test probability (PPV and NPV) confirmed the significative discrepancy between females and males being correctly classified to the proper sex group, but the high NPV rate (89%) demonstrated that the ANN enabled a very low risk of false negatives. In summary, female subjects showed a good likelihood of being correctly classified as female but provided many false positives (sensitivity = 94% and PPV = 78%), while the “male” outcome was affected by a very low risk of false negatives (NPV = 89%) although the capability of correctly classify males was lower compared to females (Sp = 68%). The same trend was found by Oliva et al. [30], Yong et al., [25], and in most of the previous studies which applied metric methods (Table 3, n. 1–6). On the contrary, very few studies [13,14] found a greater accuracy for males, suggesting that female dental traits tend to be more recognizable than male ones, generating a greater risk of error in the classification accuracy of males.

Since no similar studies on GMA applied to several dental elements for sex estimation are available in the literature, the results obtained in this research were compared to previous odontometric studies based on multiple teeth functions (Table 3). The overall accuracy yielded here (82%) appears to be quite promising in comparison with metric studies of premolars combined with molars or with other teeth (canines) (Table 3, n. 1, 3–4, 6, 8). In fact, similar performances in terms of accuracy are gained only by methods which consider both the linear and/or angular measurements of all the maxillary teeth or the molars of both jaws (Table 3, n. 2, 7). Only a few studies reported a higher accuracy considering all the variables of the whole mandibular and maxillary teeth (Table 3, n. 2, 5). In particular, Acharya et al., (2007) obtained an accuracy value of 92.5% by measuring all the mandibular and maxillary teeth on 123 dental casts, and then the same author (Acharya et al., 2011) reported an accuracy equal to 100% on a sample of 105 casts applying a logistic regression analysis to all the variables measured in all teeth.

Similarly, the sensitivity rate for females (94%) obtained in this study is significatively higher than that of most metric methods using all the variables of all teeth or maxillary groups of teeth (Table 3, n. 1–8), whilst it is comparable with Acharya et al., (2007) using all the mandibular and maxillary teeth. In the study of Acharya et al., (2011), the sensitivity for females is reported as 100% when only applying a logistic regression analysis on complete sets of dentitions instead of a discriminant analysis (most commonly used in metric measurements for sex classification), but the sensitivity is widely lower on an incomplete dentition (88.5%).

It should be highlighted that none of these studies offered a “set-aside” approach to assess the accuracy of the applied technique, as proposed in Oliva’s method. Therefore, the real performance of such methods applied to a sample different from the one used to develop the metric functions may be lower. Moreover, the accuracy decreased significatively when smaller sets of teeth were considered (Table 3, n. 1–8) and therefore they are less reliable in cases of missing teeth compared to morphometric methods.

On one hand, these findings confirmed the reliability of this morphometric method compared to previous studies on metric measurements; on the other hand, while the accuracy of metric methods decreases considerably when smaller groups of teeth are analysed, the use of GMA seems to be largely unaffected by the amount of dental elements analysed but more related to the quality of the morphological dental traits.

Taking into account the ancestry of the sample, it should be noticed that Yong et al., (Table 3, n. 9) obtained a higher performance of GMA when it was applied to the premolars of indigenous Australians compared to European Australians (Caucasian samples) and they concluded that premolars were not endowed with significant variations between the sexes but rather between the two ancestral groups. Our study, based on Caucasian Italian samples, gained higher sensitivity and specificity rates, especially in comparison with the European Australians studied by Yong (Table 3, n. 9). These findings seem to suggest an ancestry influence on dental sexual dimorphism, at least for the crown morphology of premolars.

In conclusion, the method seems to be a valuable tool for sex estimation in those forensic cases where the most common sexual dimorphic substrates, such as pelvic bones and DNA, are not available or are too damaged to perform a conclusive sex identification, e.g., in mass disasters, in cases of isolated skull or jaw recovery, in charred bodies, and in young pre-puberal skeletons [1,7,8]. Since dental evidence is easily available even in the presence of largely compromised skeletal remains, this method could be useful as a non-invasive technique, and also for archaeological findings [6].

## 5. Limitations

The main limitation of this study is the small sample size. Although the method has been validated using a specific approach to assess its reliability on an unknown population, the number used for the algorithm training and for its accuracy testing is limited and the results presented here must be considered with caution. Furthermore, the small sample size does not allow for the complete automation of the landmarks and semi-landmarks placement process, which is operator-dependent.

Another limitation is the homogeneity of the sample in relation to ancestry, whose influence on the sexual dimorphism of dental morphologies cannot be considered here, and the actual applicability of the method is only indicated for a population with the same characteristics as the training sample.

The method showed less accuracy in correctly classifying males than females, but this issue seems more related to the morphological characteristics of male teeth rather than the small size of the sample. In fact, this result confirms the observations obtained in the pilot study by Oliva et al. [30] and by Yong et al. [25], and the same trend has also been detected in most of the previous literature in which two-dimensional odontometric methods are studied (Table 3). In general, the classification of males classification by teeth seems to feature a greater risk of error than for females one.

## 6. Conclusions

The study experimented with the combined GMA–ANN method based on upper first premolar traits, as proposed by Oliva et al. (2021) [30] for the sex classification of individuals, on a larger sample and pattern of teeth. The upper premolars and first molar were considered, thus combining several predictors towards a supposedly increasing accuracy of sex estimation based on teeth morphology. The proposed approach was confirmed to be endowed with high repeatability and demonstrated that the premolars have a higher shape variation between male and female subjects. The first upper molar was characterized by less sexual dimorphic variation. The accuracy was higher (82%) than for odontometric methods that similarly consider multiple teeth. Therefore, the morphometric method appears to be more related to the quality of the sexual dimorphism of dental traits rather than to the number of the considered teeth, contrary to the classic odontometric methods.

In general, these results contribute to an increase in the scientific evidence that supports the implementation of methods based on GMA and ANN in forensic identification to be used as an alternative to classical metric methods, whose validity and applicability is highly dependent on large or complete dentitions.

Future research should investigate possible approaches based on deep learning aiming at the standardization of the positioning of landmarks and semilandmarks and the automation of the morphometric analysis, thus minimizing the influence of operator subjectivity. Furthermore, future research should verify the present evidence in samples with different ancestries and in samples of different ancient populations.

## Figures and Tables

**Table 1:** Confusion matrix of the test sample.

Reference				Parameter	Numeric Value
**Prediction**		Female	Male	Accuracy (Acc)	0.8214
	Female	29	8	Sensitivity (Se)	0.9355
	Male	2	17	Specificity (Sp)	0.6800
	“Positive” class: FEMALE			Positive predictive value (PPV)	0.7838
				Negative predictive value (NPV)	0.8947
				95% CI	(0.696, 0.9109)
				No information rate	0.5536
				*p*-value (ACC > NIR)	2.302 × 10^−5^
				Kappa	0.6301
				McNemar’s test *p*-value	0.1138
				Prevalence	0.5536
				detection rate	0.5179
				Detection Prevalence	0.6607
				Balanced Accuracy	0.8077

**Table 2:** Twenty significant principal components of the three different teeth. SP: second premolar; FP: first premolar; cs: centroid size; M: molar.

Tooth	Numerical Principal Components	Overall
Second premolar (SP)	004	100.00
First premolar (FP)	008	93.16
First premolar	004	76.73
Second premolar	002	75.89
Second premolar	013	72.72
First premolar	003	70.04
First premolar	006	63.67
First premolar	005	62.31
First premolar	Centroid size	60.68
Second premolar	001	59.43
Molar (M)	005	53.63
First premolar	012	41.71
First premolar	009	40.91
Molar	010	39.00
Molar	006	37.54
Molar	004	36.62
Molar	020	33.66
Molar	008	32.83
Second premolar	Centroid size	32.22
Molar	017	32.18

**Table 3:** Odontometric methods (1–8) and morphometric dental methods (9–10) for sex classification. Acc: accuracy results of metric functions; Se: sensitivity; Sp: specificity; post-maxilla: posterior mandibular teeth (lower first and second premolars and first and second molars); post- maxilla: posterior maxillary teeth (upper first and second premolars and first and second molars); BL: bucco-lingual measures; MD: mesio-distal measures; MBDL: mesiobuccal–distolingual cervical diameter; DBML: mesiolingual–distobuccal cervical diameter; UP1: first upper premolar; UP2: secondo upper premolar; LP1: first lower premolar; LP2: second lower premolar; Se: sensitivity; Sp: specificity; VPP; predictive positive value; NPV: predictive negative value; DA: discriminant analysis; LRA: logistic regression analysis.

*N.*	Authors/Year	Dental Method	Teeth Model	Sample	Validation	Accuracy Results of Metric Functions	Se (F) and Sp (M)
**1**	Işcan et al., 2003 [13]	Odontometric method: BL	All maxillary and mandibular left teeth (third molars excluded)	100 casts (50 F/50 M)	Cross-validation test	All variables 76%	F 80% M 72%
						All maxilla 75%	F 84% M 66%
						All mandible 74%	F 74% M 74%
						Post-maxilla 74%	F 82% M 66%
						Post-mandible 73%	F 74% M 72%
**2**	Acharya et al., 2007 [15]	Odontometric method: BL and MD	All maxillary and mandibular teeth (third molars excluded)	123 casts (58 F/65 M)	Cross-validation test	All variables 92.5%	F 95.5% M 90.3%
						All maxilla 88.7%	F 90.9% M 87.1%
						Post-mandible	
						+ all maxilla 81%	F 72.7% M 74.2%
						All mandible 79.2%	F 72.7% M 83.9%
						Post-maxilla 67.9%	F 68.2% M 67.7%
**3**	Acharya et al., 2008 [16]	Odontometric method: BL and MD	All maxillary and mandibular teeth (third molars excluded)	53 casts (22 F/31 M)	Cross-validation test	All variables (BL) 64.2%	F 68.2% M 61.3%
						All maxilla (BL) 62.3%	F 59.1% M 64.5% F
						All mandible (BL) 64.2%	68.2% M 61.3%
						All variables (MD) 83%	F 86.4% M 80.6%
						All maxilla (MD) 77.4%	F 68.2% M 83.9%
						All mandible (MD) 77.4%	F 77.3% M 77.4%
**4**	Prabhu et al., 2009 [17]	Odontometric method: BL and MD	All maxillary and mandibular teeth (third molars excluded)	105 casts (52 F/53 M)	Cross-validation test	All variables 74.3%	F 73.6% M 75%
						All maxilla 62.9%	F 62.3% M 63.5%
						All mandible 75.2%	F 75.5% M 75%
**5**	Acharya et al., 2011 [18]	Odontometric method: BL and MD	All maxillary and mandibular teeth (third molars excluded)	105 casts (52 F/53 M)	Cross-validation test	All variables (DA) 57.1%	F 55.8% M 58.5%
						All maxilla (DA) 52.4%	F 48.1% M 56.6%
						All mandible (DA) 70.5%	F 69.2% M 71.1%
						All variables (LRA) 100%	F 100% M 100%
						All maxilla (LRA) 76.2%	F 76.9% M 75.5%
						All mandible (LRA) 84.8%	F 82.7% M 86.8%
**6**	Mujib et al., 2014 [20]	Odontometric method: MBDL and DBML	Maxillary canines and first molars	100 casts (50 F/50 M)	/	All variables 71%	F 73% M 69%
						All canines 67%	F 66% M 67%
						All molars 65%	F 66% M 65%
**7**	Kazzazi et al., 2017 [23]	Odontometric method: BL and MD	Maxillary and mandibular molars (third molars excluded)	75 subjects (28 F/52 M)	Cross-validation test	All maxillary I molars 82.1%	F 69.2% M 90.2%
						All maxillary II molars 85.5%	F 82.6% M 87.2%
						All mandibular I molars 78.4%	F 64.3% M 87%
						All mandibular II molars 83.5%	F 70.4% M 90.4%
**8**	Tabasum et al., 2017 [24]	Odontometric method: MBDL, DBML, MD, and BL	Maxillary and mandibular molars	130 subjects (73 M/57 F)	/	All maxillary molars MD (DA) 67%	F 75.9% M 58.6%
						All maxillary molars BL (DA) 67%	F 65.5% M 69%
						All maxillary molars MD (LRA) 67%	F 75.9% M 58.6%
						All maxillary molars BL (LRA) 67%	F 65.5% M 69%
**9**	Yong et al., 2018 [25]	GMA	UP1, UP2, LP1, LP2	140 casts (70 F/70 M)	Cross-validation test	UP1 Indigenous Australians/	F 74.3% M 80%
						UP2 Indigenous Australians/	F 80% M 74.3%
						UP2 European Australians/	F 62.9% M 57.1%
						UP1 European Australians/	F 57.1% M 57%
**10**	Oliva et al., 2021 [30]	GMA + ANN	UP1	100 scans (50 F/50 M)	Training sample (75 scans) and test sample (25 scans)	Training sample 84%	F 92% M 70%
						Test sample 80%	VPP F 90% M 73%
							NPV F 73% M 90%

## Data Availability

Not applicable.
